# Reflections on Several Landmark Advances in Circadian Biology

**DOI:** 10.5334/jcr.236

**Published:** 2024-04-01

**Authors:** Sangeeta Chawla, Henrik Oster, Giles E. Duffield, Erik Maronde, Mario E. Guido, Christopher Chabot, Ouria Dkhissi-Benyahya, Ignacio Provencio, Namni Goel, Shawn D. Youngstedt, Natalie Zi-Ching Mak, Mario Caba, Anjoom Nikhat, Shaon Chakrabarti, Lei Wang, Seth J. Davis

**Affiliations:** 1 Department of Biology, University of York, York YO105DD, UK; 2 Institute of Neurobiology, Center for Brain, Behavior & Metabolism (CBBM), University of Luebeck, 23562 Luebeck, DE; 3 Department of Biological Sciences and Eck Institute for Global Health, Galvin Life Science Center, University of Notre Dame, Notre Dame, IN 46556, US; 4 Institut für Anatomie II, Dr. Senckenbergische Anatomie, Goethe-Universität Frankfurt, Theodor-Stern-Kai-7, 60590 Frankfurt, DE; 5 CIQUIBIC-CONICET, Facultad de Ciencias Químicas, Universidad Nacional de Córdoba, Córdoba, AR; 6 Departamento de Química Biológica Ranwel Caputto, Facultad de Ciencias Químicas, Universidad Nacional de Córdoba, Córdoba, AR; 7 Department of Biological Sciences, Plymouth State University, Plymouth, NH 03264, US; 8 Inserm, Stem Cell and Brain Research Institute U1208, Univ Lyon, UniversitéClaude Bernard Lyon 1, 18 Avenue du Doyen Lépine, 69500, Bron, FR; 9 Department of Biology and Department of Ophthalmology, University of Virginia, Charlottesville, VA, US; 10 Biological Rhythms Research Laboratory, Department of Psychiatry and Behavioral Sciences, Rush University Medical Center, Chicago, IL, US; 11 Edson College of Nursing and Health Innovation, Arizona State University, Phoenix, AZ, US; 12 Department of Medicine, University of Arizona, Tucson, AZ, US; 13 Centro de Investigaciones Biomédicas, Universidad Veracruzana, Xalapa, Ver., MX; 14 Simons Centre for the Study of Living Machines, National Centre for Biological Sciences, Bangalore, Karnataka 560065, IN; 15 Key Laboratory of Plant Molecular Physiology, CAS Center for Excellence in Molecular Plant Sciences, China National Botanical Garden, Beijing 100093, CN; 16 State Key Laboratory of Crop Stress Biology, School of Life Sciences, Henan University, Kaifeng 475004, CN

**Keywords:** Circadian, Clock genes, Entrainment, Master Clock, Luciferase, Retina, Photoperiod, Metabolic oscillator, Crop plants, Coupled oscillator, Food-entrainable oscillator, Chronotherapy

## Abstract

Circadian Biology intersects with diverse scientific domains, intricately woven into the fabric of organismal physiology and behavior. The rhythmic orchestration of life by the circadian clock serves as a focal point for researchers across disciplines. This retrospective examination delves into several of the scientific milestones that have fundamentally shaped our contemporary understanding of circadian rhythms. From deciphering the complexities of clock genes at a cellular level to exploring the nuances of coupled oscillators in whole organism responses to stimuli. The field has undergone significant evolution lately guided by genetics approaches. Our exploration here considers key moments in the circadian-research landscape, elucidating the trajectory of this discipline with a keen eye on scientific advancements and paradigm shifts.

## Introduction

Over the past 15 to 30 years, circadian research has witnessed an extraordinary evolution, marked by groundbreaking scientific advances. The identification of “clock genes” in fruit flies, plants, bacteria, fungi, and mammals, a feat accomplished first by researchers like Ron Konopka and Seymour Benzer, paved the way for unraveling the molecular underpinnings of circadian timekeeping. The elucidation of gene expression feedback loops, coupled with the development of luciferase reporters, ushered in a new era of monitoring transcriptional and translational rhythms in living organisms. The realization that multicellular organisms house multiple clocks, rather than a singular central pacemaker, reshaped our understanding of internal temporal synchronization. The discovery of intrinsically photosensitive retinal ganglion cells (ipRGCs) and their role in circadian photoentrainment added a new dimension to our comprehension of photoreception. Coupled oscillators, both theoretically and physically conceptualized, provided a unified framework, offering insights into how endogenous oscillators synchronize with environmental cycles.

These reflections on the historical milestones and current insights underscore the interdisciplinary nature of circadian biology. As we stand on the shoulders of past achievements, the significance of identifying clock genes, deciphering gene expression feedback loops, and understanding the role of nonphotic cues becomes evident. This journey through time not only highlights the resilience of early scientific excitement but also serves as a compass for future circadian explorations. The intricate interplay of theoretical concepts, molecular research, and practical applications demonstrates the far-reaching implications of circadian biology, touching realms from agriculture to human health. Through these reflections, we aspire to inspire the next generation of circadian scientists, encouraging them to unravel the remaining mysteries and propel circadian research into new frontiers.

**Gene hunting**

Sangeeta Chawla, Henrik Oster, Giles E. Duffield, Erik Maronde

The endogenous nature of circadian rhythms, inferred from their persistence in constant conditions, had been known for centuries. A key breakthrough in chronobiology was the identification of “clock genes” in the late 20^th^ century, first in flies and then in mammals, eventually leading to the elucidation of gene expression feedback loops as the mechanistic basis for circadian timekeeping. The era of clock genes began with the seminal work of Ron Konopka and Seymour Benzer who screened mutant flies for altered circadian behaviours to identify the *per* mutants in 1971 [1]. Indeed, Seymour Benzer is credited with founding the field of neurogenetics. The idea that a single gene can control behaviour [2] was met with scepticism when Seymour Benzer first embarked on his work on fruit flies in the late 1960s. Remarkably, Benzer and his colleagues isolated mutants with a range of behavioural deficits, some of these with ingenious bespoke apparatus [3]. The circadian rhythm mutant screen, for example, used an eclosion monitor to measure rhythms in the emergence of flies from pupae and infrared light beams to measure locomotor activity rhythms. Konopka and Benzer isolated not just circadian arrhythmic mutants but also those with shorter (19 h) and longer rhythms (28 h) that mapped to the same gene. It took several years to appreciate the significance of these shorter and longer mutations as seminal work done over the next two decades by Rosbash, Young, and Hall revealed the interactions of the Drosophila period protein [4,5] with additional clock genes including timeless [6,7] and how these genes and post-translational regulation of their gene products generated gene expression loops [8,9] with 24 h periodicity.

The discovery of insect clock genes fuelled a search for “clock genes” in mammals. In the 1990s, many labs were hunting for and discovering the mammalian homologues of the fly clock genes that Benzer, Rosbash, Hall, and Young had discovered in the 1970s and 1980s. In 1997 Joe Takahashi’s group cloned “CLOCK” [10] and the mammalian period genes were described almost simultaneously by the Okamura [11,12], Reppert [13] and Lee labs [14], although in the Lee lab they initially named it Rigui (after a Chinese sundial). Sequence homologies between mammals and flies were rather moderate which made it difficult to identify homologues (in the absence of fully annotated genome sequences at the time). Some confusion assumed with regard to the dTim homologue Timeless – which turned out to be a false friend and not related to circadian clock function at all [15].

The initial cloning and characterization of the mammalian period (per) genes, revealed in studies published between 1997 and 2000 was a landmark achievement that transformed the landscape of chronobiology and the understanding of the mammalian circadian clock. Not only did it open up the molecular black box of the hypothalamic suprachiasmatic nucleus (SCN) clock but also shifted perspective on the existence and character of the peripheral clock [16] and bolstering the concept of the cell autonomous clock [17] in the mammal. The discovery of the period 1 (per1) gene was followed quickly thereafter by identification of per2 and per3, also known as mPer1, mPer2 and mPer3, ‘m’ as in ‘mouse’ [13,18,19,20]. Key findings were that all were expressed within the SCN, and later revealed to be present in many tissues across the mouse and rat body, were rhythmically expressed at mRNA level with an approximate 24-hour periodicity. The important feature of the period genes is that not only are per1 and per2 state variables of the clock, but that their levels can be induced by light/photic-signals [12,21], while being suppressed by non-photic behavioural arousal entrainment cues [22]. They are immediate early genes (IEGs), not requiring any prior *de novo* protein synthesis to induce their gene expression. They are the molecular gateway into the resetting of the circadian clock. The period genes are critical components of the clock, forming the negative portion of the transcriptional-translational feedback mechanism that comprises the molecular clock. The paired genetic deletion of per1 and per2 combined renders the clock non-functional [23]. The revelation that a single polymorphism of the per2 gene in the human could result in such a profound phenotype as familial advanced sleep phase syndrome with a ~4-hour phase angle difference [24] cemented the role of this gene family in shaping the function of the circadian timing machinery in the human. After the core circadian clock loop with Clock, Bmal1, Periods, and Cryptochromes had been described, the accessory loops were discovered by the Schibler [25] and Honma labs [26], which ended the golden age of clock gene hunting. Several modulators have been described and genes linking clock function to physiology, but the field quickly moved on to studying the functional consequences of clock disruption. The available knockout mice with their multiple and complex phenotypes continue to create interesting research questions even today.

The knowledge of genes and gene loops in flies and mammals allowed for development and utilisation of reporters that could be used to monitor transcriptional and translational rhythms in living cells, organs and organisms.

**Studying the clock in real time**

Seth J. Davis

One of the greatest technical innovations in chronobiology is the transgenic introduction of luciferase as a vital reporter to indirectly measure transcription for days in living cells and organisms. The first report of this was the introduction of firefly luciferase to the model plant Arabidopsis [27]. Soon after this, a bacterial luciferase was shown to drive similar rhythms in cyanobacteria [28]. Using the firefly system, the first ever circadian mutants in plants were found and characterised [29,30]. This system has also been used to perform numerous physiological studies in Arabidopsis, and other plants. It was used in quantitative genetics studies to reveal natural allelic variation in rhythm patterns in the lab [31] and in the field [32,33]. These Arabidopsis findings led to similar capacity to explore the firefly system in animals, where it was first used in *Drosophila* [34] and then living mouse explants [35].

Overall the pioneering use of firefly luciferase in Arabidopsis had profound implications to the whole of the circadian clock community, with implications far beyond plant studies and a key finding enabled by this technology was how pervasive cellular clocks are.

**Incorporating a Metabolic Oscillator**

Mario Eduardo Guido

Beyond the canonical transcription–translation-based feedback loop the groundbreaking discovery of a ‘metabolic oscillator’ added another mechanistic dimension to cellular timekeeping [36,37,38]. The metabolic oscillator drives rhythms in the cellular redox state generating oscillations in reactive oxygen species, peroxiredoxin oxidation state, glycerolipid enzyme expression, metabolism and energy store, among other parameters [39]. These metabolic oscillations can be observed in normal cells and tissues and even in proliferating tumor cells (glioma, hepatocellular carcinoma, etc.) after synchronization, and such oscillations still persist in the absence of transcription as seen in enucleated cells red blood cells [40,41,42]. The discovery of a metabolic oscillator has raised questions about its role in cellular time-keeping, its relationship with the TTFL-based molecular clock, and the implications of this in scenarios where the circadian system is disrupted as a consequence of modern life by factors such as continuous artificial illumination, nocturnal shift work, jet lag, hypocaloric diets and sedentary life amongst others [36,37].

**Clocks everywhere: Beyond the masterclock**

Christopher C. Chabot

A key landmark paper from the Schibler lab [17] demonstrated that multicellular organisms are composed of many clocks, not just one central pacemaker. Cited nearly 1700 times since its publication (Web of Science, 2024), this research paved the way towards an understanding that internal temporal synchronization involved not just temporal control by a master clock but also among many clocks. A desynchronization of these multiple clocks is thought to underlie many of the problems associated with shift work in humans, including some types of depression and bipolar disorders. Importantly, these shift workers, who comprise approximately 20% of the US workforce [43], also have significantly increased rates of obesity [44], cancer, heart disease [45] and other metabolic issues [46]. These findings also spawned hundreds of investigations of the importance of this internal synchronization in humans and other animals and has helped to provide important data for the new field of chronotherapy [47].

An important example of how cell autonomous clocks come together at an organ level is exemplified by the peripheral clock of the retina.

**The retina: a unique model of circadian clock**

Ouria Dkhissi-Benyahya

The retina has helped us understand how cellular clocks are organised in a hierarchical manner to generate a tissue clock. One of the first rhythms described in the mammalian retina was the cycle of rod outer segment disc shedding with lighting conditions [48]. Later, experiments performed in amphibians and birds demonstrated that the retina contains an endogenous circadian clock, able to oscillate in constant condition in a culture dish [49,50]. However, it was not until 1996 that cultured hamster retina then mouse retina was reported to maintain autonomous circadian rhythms in melatonin secretion, providing evidence that an endogenous clock is also present in the mammalian retina [51,52]. Based on molecular and physiological data from amphibians and birds, the initial prevailing model for circadian organisation in vertebrate retinas proposed that photoreceptors are the primary site of rhythm generation. By contrast, the exact location of the retinal clock in mammals has been a matter of long debate since its discovery in 1996. Using clock gene/protein expression and bioluminescence recording with luciferase reporter coupled to clock genes, several studies came to the consensus that retinal neurons as well as glial cells express the molecular clock machinery (for review, see [53]. Additional evidence demonstrated that the regulation of rhythmicity in the mammalian retina proceeds from a network of strongly coupled oscillators located within distinct cellular layers [54,55]. The mammalian retina thus constitutes a fascinating clock model perfectly suitable to both understand the molecular mechanisms of circadian rhythm generation at the cellular and tissue level and to characterise synchronising factors coordinating multiple oscillators at the tissue level.

Groundbreaking research featured the retina in the seminal discovery of intrinsically photosensitive melanopsin retinal ganglion cells to shed light on novel aspects of photoreception and how photic stimuli contribute to circadian entrainment. Recent research, however, contends that neuropsin and/or rods, rather than melanopsin retinal ganglion cells, are necessary for the light entrainment of the mammalian retinal clock [56,57].

**Time for Entrainment**

Ignacio Provencio, Namni Goel, Shawn D. Youngstedt, Natalie Zi-Ching Mak and Mario Caba

In the 1980s, evidence began to emerge that animals with retinal degeneration still could entrain locomotor rhythms to light:dark cycles [58,59] Later it was shown that non-visual responses to light even persisted in blind humans [60]. Importantly, bilateral surgical removal of the eyes renders mammals incapable of photoentraining their activity rhythms [61]. Taken together, these findings suggested that while the eyes are necessary for photoentrainment in mammals, rod and cone photoreceptors may be dispensable. These results paved the way for the search of a non-rod, non-cone ocular photoreceptor class underlying light’s impact on the circadian axis. In 2002, David Berson and colleagues identified intrinsically photosensitive retinal ganglion cells (ipRGCs), a type of retinal photoreceptor not previously known to exist [62]. ipRGCs project to the suprachiasmatic nucleus, the master circadian oscillator that governs circadian activity rhythms, and provide a neural pathway by which the central circadian pacemaker may be reset by light. The discovery of ipRGCs spawned a cottage industry of labs dedicated to understanding the signaling, anatomy, physiology, and function of these unique photoreceptors critical for circadian photoentrainment [63].

In parallel, studies on how nonphotic stimuli influence the circadian system in rodent models revealed how light exposure and wheel running could have synergistic or antagonistic effects on the circadian system depending on the timing of exposure to these zeitgebers. Particularly influential was the research by Mrosovsky et al. [64,65,66] and Mistlberger et al. [67,68,69]. Their pioneering studies inspired a series of human studies by the Youngstedt lab and others [70,71,72,73], including an ongoing study examining whether there are additive effects of bright light, exercise, and melatonin for facilitating humans to adjust to simulated jet lag [74].

The integration of photic and nonphotic stimuli effects (and their potent interactions) on the circadian system highlighted a clear necessity for the development of diurnal rodent models to allow extrapolation and comparisons of these circadian effects to those emerging in humans. These needs inspired the work by Goel et al. (e.g., [75,76,77,78,79,80]) and that of others on the broad behavioral and neuroanatomical characterization of photic and nonphotic stimuli on the circadian system and their mechanisms in a diurnal species. This work informed the development of cues to phase shift and modify the human circadian system in clinical and nonclinical settings.

A much studied nonphotic cue is food. When food is restricted to specific hours during the day, after several days, animals develop intense preprandial locomotor activity and general arousal, which is termed food anticipatory activity (FAA; [81]). It had been considered that FAA is controlled by a putative food entrainable oscillator (s) (FEO; [82]), but their locus, if any at central and/or peripheral level, remains elusive. FAA had been intensely studied in several laboratory species including rabbits [83,84], but most research had been performed in rodents [85]. However, even in a single species such as rats, there are a wide array of differences in the definition, experimental approach and interpretation of data, which make it more difficult to draw conclusions about the FEO. FAA had been studied for around 100 years in laboratory conditions. Over this period much information had been gathered about food and circadian physiology [86] and even the interaction between central and peripheral structures [87]. The interest to study this phenomenon has not decreased mainly because it suggests the existence of a strong oscillator(s) entrained by food distinct from that of the main circadian oscillator entrained by light in mammals. It is an open and exciting field of research for the new generation.

**Coupling oscillators**

Anjoom Nikhat and Shaon Chakrabarti

Circadian biology is one of the areas where theoretical and physical concepts have truly provided a fundamental unifying conceptual framework. While 24 hour rhythms in physiology had been appreciated by humans from ancient times, it was not until the late 20^th^ century that it got firmly established that such rhythms are not driven by the daily cycles of day and night, but are a result of synchronization of endogenous oscillators in organisms to the environmental cycle [88,89]. Just as two physical pendulums with somewhat different time-periods eventually exhibit oscillations with a common frequency after coupling via a wooden beam, one can conceptualize the effect of the day-night cycles as entraining existing biological rhythms within organisms. To a large extent this pivotal conceptual advance in the field of circadian clocks was driven by research in the theory of dynamical systems, and led to exciting advances in the late 1900’s and early 2000’s, particularly in the field of coupled oscillators [90,91,92]. The puzzling earlier observations on the variability of free-running oscillation time periods across organisms could be explained quantitatively, as could the existence of a wide range of human chronotypes and different phase response curves [93,94]. Ideas such as entrainment range and phase of entrainment that were only qualitatively understood till then, could be related to the properties of the two coupled oscillators involved [95,96]. These advances in turn spurred efforts to understand at a molecular level, how various external “zeitgebers” could reset the clocks within organisms, and how these clocks (de)synchronize with each other in health and disease [97,98,99]. In retrospect, the innate or endogenous nature of clock oscillations which we almost take for granted now, is a fundamentally important property, without which none of the current areas of circadian clock research would even exist.

**The time is ripe**

Lei Wang

The knowledge of how clock mechanisms relate to physiology has huge potential applications and one such field of translation is in improving human use of crops.

Circadian clocks are evolved to facilitate crops to adapt the daily and seasonally changed environment cues such as light and temperature. Soybean, maize, wheat and rice are staple crop in many of countries. The proper flowering time and maturity are vital for their domestication from the origin place to the lower or higher latitudes respectively. Recently, many of key genetic factors involved into their domestication were manifested to be core circadian components. For example, soybean, the facultative short day plant, originated from the temperate regions of China. Soybean cultivars with the dysfunction of the homologs of Arabidopsis Pseudo Response Regulators were preferably selected to ensure the early flowering time and maturity in the high latitude regions with longer day length [100]. By contrast, deficient of soybean homolog of LATE ELONGATED HYPOCOTYL (LHY) and Early Flowering 3 (ELF3), which both are core components of Evening Complex, was genetically targeted to adapt to the low latitude regions to avoid prematurity and to achieve high yield [101,102,103]. In wheat, the natural variations in *Ppd-1* gene, one wheat homolog of PRR gene family, not only affect the photoperiod sensitivity, but also affect important agronomic traits, including plant height and grain weight [104,105]. Similarly, rice Days to heading 7 (DTH7)/OsPRR37 is also reported as a major determinant for grain yield and photosensitivity [106,107]. Besides, core clock components were also shown to be major player for conferring abiotic stress tolerance and for controlling key developmental processes in crops [108,109,110,111]. Therefore, the knowledge of circadian systems in crops will further facilitate molecular design to construct superior cultivars to better adapt the local environments, in particular, for helping the *de novo* domestication of the new valuable plant resource.

## Concluding remarks

In conclusion, our reflective journey through the annals of circadian biology illuminates the multifaceted nature of this dynamic field. From the pioneering identification of clock genes to the intricate dance of coupled oscillators, the historical milestones have not only shaped our present understanding but also cast a guiding light toward future endeavors. Circadian biology has transcended the confines of temporal regulation, intertwining with diverse aspects of life, from the molecular level to agricultural practices and human health. The realization that organisms harbor multiple clocks and the discovery of peripheral clocks underscore the complexity of internal temporal synchronization, providing crucial insights into health-related challenges arising from desynchronization.

Reflecting on the past serves as more than just a nostalgic exercise; it becomes a call for action. As we stand on the cusp of the next wave of circadian research, the lessons from historical breakthroughs beckon us to explore new horizons. The integration of theoretical concepts with molecular research has set the stage for experiments that delve into the intersections of circadian biology with emerging fields. Leveraging the knowledge gained from clock genes, coupled oscillators, and the impact of nonphotic cues, we are poised to embark on experiments that bridge the gap between historical literature and contemporary trends. The challenge lies in unlocking the full potential of circadian biology, not only to deepen our understanding of biological timing but also to pioneer innovative applications that can revolutionize agriculture, human health, and beyond. As we look to the future, the echoes of the past resonate, urging us to unravel the remaining mysteries and inspire the next generation of circadian scientists to embark on their own transformative journeys.
